# Adenotonsillotomy versus adenotonsillectomy in pediatric obstructive sleep apnea: A 5-year RCT

**DOI:** 10.1016/j.sleepx.2022.100055

**Published:** 2022-09-08

**Authors:** Isabella Sjölander, Anna Borgström, Pia Nerfeldt, Danielle Friberg

**Affiliations:** a Department of Surgical Sciences, Uppsala University, Uppsala, Sweden; b Department of Clinical Science, Intervention and Technology, CLINTEC, Karolinska Institutet, Stockholm, Sweden

**Keywords:** ATE, adenotonsillectomy, ATT, adenotonsillotomy, OSA, obstructive sleep apnea, OSDB, obstructive sleep-disordered breathing

## Abstract

**Objectives:**

Adenotonsillectomy (ATE) is a common treatment for pediatric obstructive sleep apnea (OSA). Intracapsular adenotonsillotomy (ATT) is associated with less postoperative morbidity. Our previous randomized controlled trial (RCT) compared ATE and ATT in otherwise healthy children with moderate to severe OSA. No differences in polysomnographic (PSG) and OSA-18 were found between the groups at one-year follow-up. This study presents the long-term results of the RCT.

**Methods:**

Non-obese children (n = 79, 2–6 years) who had undergone either ATE (n = 40) or ATT (n = 39) were offered PSG and OSA-18 questionnaire five-years after surgery. Primary outcome was the group difference in postoperative Obstructive Apnea/Hypopnea Index (OAHI). ATE was recommended to the ATT group if they had a relapse of OSA.

**Results:**

The follow-up was completed by 45 of 79 (57%) children; 28 (35%) drop-outs, and six of 39(15%) in the ATT group were excluded after ATE. After ATE(n = 17), OAHI decreased from mean 12.3(SD 8.0) to 0.6(0.7), and after ATT(n = 28) from 12.6(7.4) to 0.5(0.6), a mean difference in postoperative OAHI of 0.1(95% CI -0.3 – 0.5). Sensitivity analyses did not change the results. The median OSA-18 decreased in the ATE group from 57(interquartile range 47–79) to 27(22–36), and in the ATT group from 67(53–79) to 32(25–44), without group differences for postoperative values.

**Conclusion:**

The results of this five-year follow-up of otherwise healthy OSA-children showed a high drop-out rate, but indicates that ATT could be an effective treatment for pediatric OSA. However, ATT warrants follow-up due to the risk of recurrence, and further studies are needed.

## Highlights

• Adenotonsillotomy is an effective treatment for Obstructive sleep apnea in otherwise healthy children.
• Six children out of 39 were reoperated with adenotonsillectomy within three years.
• Follow-up after adenotonsillotomy is recommended.

## 1 Introduction

Pediatric obstructive sleep apnea (OSA) is the most severe form of obstructive sleep-disordered breathing. It occurs when breathing repeatedly pauses during sleep because of an obstruction in the upper airway. The estimated prevalence of OSA among children is 1–6% [1], with the peak between 2 and 8 years of age when tonsil and adenoid lymphoid growth is most active [2]. Hence, the most common cause of OSA in children is enlarged tonsils and adenoids, and the most common surgical treatment is to remove the tonsils, either with or without the adenoid (adenoidectomy). International guidelines recommend surgery in children with moderate to severe OSA (Apnea Hypopnea Index, AHI >5), or a high degree of OSA symptoms [3,4]. The removal of the palatine tonsils can be done by a tonsillectomy (total removal of the whole tissue including the capsule – extracapsular removal), or by a tonsillotomy, which is a less invasive surgical method where only the tonsillar tissue protruding from the anterior tonsillar pillar is removed and the capsule is preserved.

Tonsillotomy is a technique associated with less postoperative pain, a lower risk of severe hemorrhage, and a shorter period of sick leave for both the child and the caregiver [[5], [6], [7]]. However, leaving tonsillar tissue can allow the tonsils to grow back, which can sometimes require reoperation for the residual/recurrent OSA [8]. Intracapsular technique is a surgical approach that is becoming more common worldwide. It is important to scrutinize the risks and benefits of this procedure compared to standard tonsillectomy [9]. Several systematic reviews have concluded that adenotonsillotomy (ATT) is associated with less postoperative morbidity and shorter length of surgery than adenotonsillectomy (ATE) but with a similar effect on OSA symptoms [[10], [11], [12], [13], [14]]. However, there is a lack of prospective long-term follow-ups to determine the risk of regrowth and reoperation rates.

According to the 2020 Cochrane report, there are no prospective randomized studies that compare respiratory events during sleep after 12 months of ATT and ATE follow-up [9]. The report concludes that the certainty of evidence is low as to which procedure is recommendable, and there is a need for long-term studies that examine the effects of ATT and ATE.

Our original randomized controlled trial (RCT) of otherwise healthy children with OSA showed no significant differences in changes of AHI among children treated with ATE compared to ATT assessed with polysomnography and OSA-18 one year after surgery [15]. This study aims to analyze long-term follow-up results of PSG, with changes in obstructive AHI (OAHI), OSA-18, and the percentage risk of reoperation after ATT.

## 2 Material and methods

### 2.1 Study group

This prospective single-center randomized trial was conducted at Karolinska University Hospital's Oto-, rhino and laryngology department from 2011 to 2021. Seventy-nine children aged from 2 to 6 years were included. See our previous study for a detailed description of the power analysis and randomization process [15]. The original inclusion criteria were age 2–6, symptoms of OSA, tonsil hypertrophy 3 or 4 according to the Brodsky scale [16], apnea–hypopnea index (AHI) of ≥5 and ≤ 30 events/hour sleep. Exclusion criteria were craniofacial abnormality, neuromuscular disease, chromosomal abnormality, obesity (BMI z-score >1.67) [17,18], previous adenotonsillar surgery, bleeding disorder, cardiopulmonary disease, history of recurrent tonsillitis, and parents with insufficient knowledge of Swedish. After the one-year follow-up the caregivers were urged to call the clinic whether the child had signs of recurrence of OSA-symptoms.

### 2.2 Outcomes

The differences between postoperative values in the ATE and ATT groups were examined. The primary outcome was exchanged from the 1-year follow-up with changes in AHI, to this 5-year with changes in OAHI. The secondary outcomes were other PSG variables and OSA-18 score. The need for repeated tonsil surgery was also evaluated. Children who had undergone ATT were recommended reoperation with ATE if they showed signs of tonsil regrowth and recurrence of OSA symptoms, AHI ≥5 or recurrent tonsillitis. Children who were reoperated with tonsillectomy were excluded from the primary per protocol analysis but included in the sensitivity analysis.

### 2.3 Polysomnography

All children attended overnight in-laboratory PSG1 at baseline before surgical treatment, with a follow-up one year after (PSG2) and a long-term follow-up (PSG3) five years after surgical intervention. The PSGs were performed using the same EMBLA technology (Flaga Medical, Reykjavik) at the same place (Karolinska University Hospital, Huddinge, Sweden). All PSGs were scored manually by a registered polysomnographic technologist, who was blinded to participant group. The American Academy of Sleep's pediatric scoring rules were used [19]. Mild OSA was defined as OAHI ≥1 and < 5 per hour [3].

### 2.4 OSA-18 quality of life survey

The parent/caregiver completed the OSA-18 questionnaire on the same evening as each of the PSG procedure (at baseline before surgery, one-year follow-up, and five-year follow-up) [20]. OSA-18 is a validated quality of life questionnaire that assesses symptoms of OSA during the previous four weeks. The questionnaire consists of 18 items within five domains (sleep disturbance, physical suffering, emotional distress, daytime problems, and caregiver concern). The caregiver scores on a 7-point Likert scale, with the total system score ranging from 18 to 126 points. If the total OSA-18 score is 60 or greater, it indicates the presence of OSA [21]. The Swedish version of the questionnaire also includes a general question about the child's general health-related quality of life (HRQoL) in relation to their symptoms. The caregiver is asked to grade overall quality of life between 0 and 10. In this case, 0 indicates the worst possible quality of life, and 10 is the best possible quality of life.

### 2.5 Surgical intervention

Tonsillectomies were performed with blunt extracapsular dissection using cold steel. Tonsillotomies were performed by coblation (controlled ablation) where intracapsular tissue was removed to the anterior tonsillar pillars without exposing the pharyngeal muscle. Adenoidectomies were performed for all patients during the same session using cold steel (ring knife). Experienced surgeons performed all surgical interventions.

### 2.6 Ethics

This study was approved by the regional ethical board in Stockholm (Dnr 2011/925–32) with a complementary number of patients (Dnr 2013/2274–32).

### 2.7 Statistical analysis

Data were analyzed using Stata and R Studio. Continuous data from PSG-variables were presented as mean (Standard Deviation, SD) or as mean and 95% Confidence Interval, CI, and ordinal data from OSA-18 as median (interquartile range). An independent (unpaired) *t*-test was used for continuous data and Mann Whitney U (Wilcoxon two sample test) for ordinal data. A chi-squared test was used for categorical data. Per protocol analysis was performed using observed data. Sensitivity analyses of intention-to-treat were performed for the primary outcome OAHI, including dropouts from both groups and the six reoperated children in the ATT treatment group. Missing values were imputed using the last observation carried forward (LOCF). In addition, a simulated negative scenario for the ATT group was made for the primary outcome with replacement of LOCF OAHI <5 with OAHI = 5. Also, a dropout analysis was conducted comparing baseline characteristics between patients included and dropouts/excluded. The level of significance was defined as a two-sided p-value <0.05.

## 3 Results

**Fig. 1:**
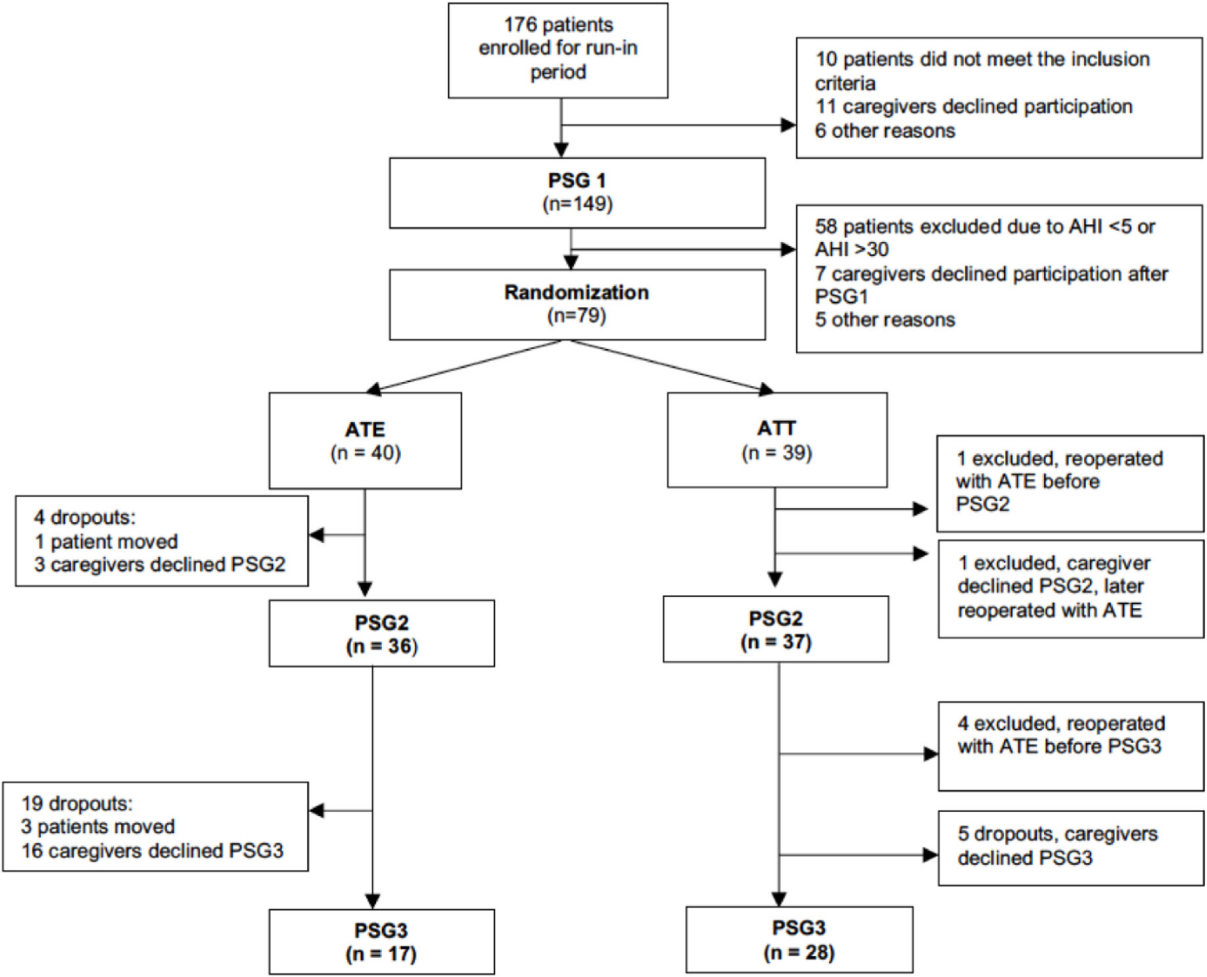
Flow chart of patients included in this study.

Of the 79 randomized patients (Table 1), 28 (35%) dropped out at the five-year follow-up. Six of 39 (15%) children from the ATT group were excluded because of reoperation with ATE. In total, 45 children (57%) were included in the per protocol analysis, with a drop-out/exclusion rate of 43%. Seventeen of 40 patients (43%) from the ATE group and 28 of 39 (72%) from the ATT group underwent PSG3 and the per protocol analysis (Fig. 1). The median age at PSG3 was nine years and six months (114 months, interquartile range 107–120).

**Table 1:** Baseline characteristics.

	ATE (n = 40)	ATT (n = 39)
Age, mo	47 (15)	45 (15)
Male sex, n(%)	29 (73)	24 (62)
Length, cm	98 (13)	99 (10)
Weight, kg	15.7 (3.1)	15.3 (3.3)
Tonsil size, 1–4 [1]	3.3 (0.6)	3.5 (0.6)
Adenoid size, 1–4	2.7 (0.8)	3.0 (0.7)
AHI	14.5 (7.3)	15.4 (7.3)
OAHI	12.5 (7.8)	13.4 (7.3)

Values are mean (SD) unless specified otherwise. ^1^Tonsil size according to Brodsky [16], occlusion of the oropharynx [%] 1: 0–25%; 2: 26–50%; 3: 51–75%; 4: 76–100%. AHI = apnea hypopnea index.

**Table 2:** Dropout analysis with comparison of baseline characteristics between children who attended 5-year follow-up and dropouts/excluded.

Parameter	Included	Dropout/Excluded	P value
n (%)	45 (57%)	34 (43%)	
Age, mo	42 (15)	45 (14)	0.3^a^
Surgical treatment ATE/ATT	17/28	23/11	0.009^b^
Sex boy/girl (%)	28/17 (62/38)	25/9 (74/26)	0.3^b^
OAHI			
Baseline	12.5 (7.5)	14.3 (7.5)	0.3^a^
1 year	1.3 (1.1)	2.5 (5.2)	0.15^a^
OSA-18, median (range)			
Baseline	65 (50–79)	63 (55–74)	0.8^c^
1 year	31 (26–39.5)	30 (23–43)	0.6^c^

Values are mean (SD) if not specified otherwise Probability calculated with ^a^t test, ^b^chi-square test, ^c^Mann Whitney U.

### 3.1 Primary outcome

**Fig. 2:**
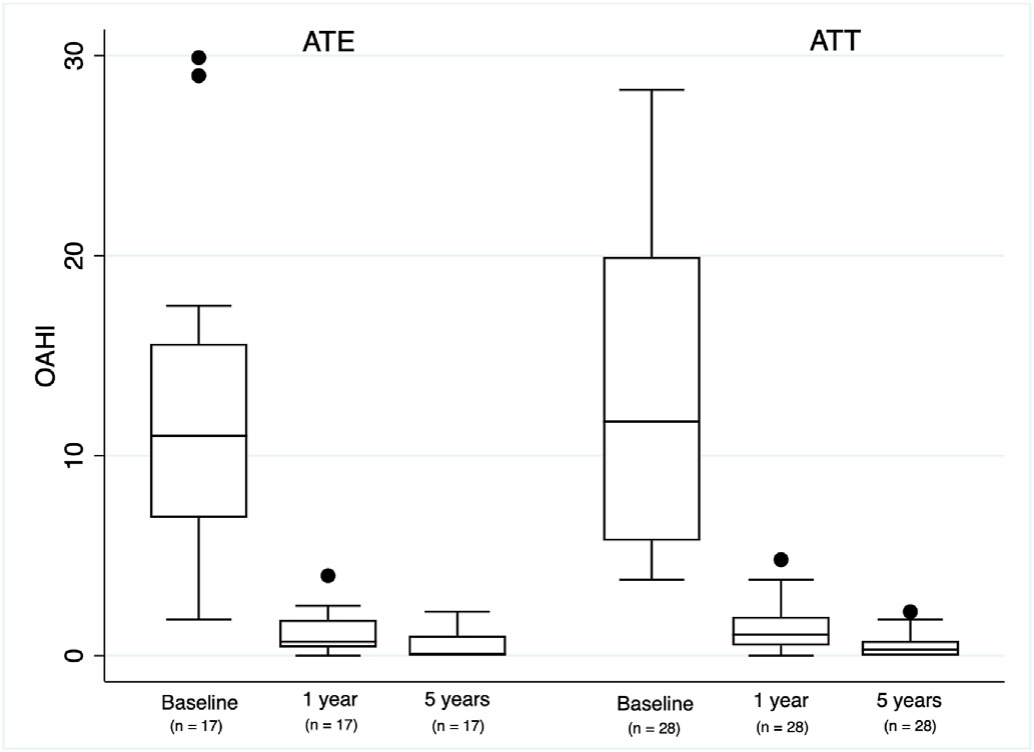
Boxplots illustrating obstructive apnea hypopnea index (OAHI) at baseline, 1-year, and 5-year follow-up for all patients included in the per protocol analysis. Boxes show the median, 25% and 75% values, whiskers show the non-outlier range and dots represent the outliers.

The mean OAHI decreased in the ATE group from baseline 12.3 (SD 8.0) to 5-year follow-up 0.6 (0.7), a 95% mean reduction, and in the ATT-group from 12.6 (7.4) to 0.5 (0.6), a 96% mean reduction (Fig. 2). The per protocol analysis showed a mean difference between the groups postoperatively with 0.1 (95% CI -0.3 – 0.5). Five children in each group had mild OSA (OAHI ≥1-<5), the rest had OAHI <1. For all PSG results, see Table 3.

**Table 3:** Results from PSG and OSA-18 variables – baseline (PSG1), one year (PSG2), and five years (PSG3) in the per protocol analysis.

	ATE PSG1 (n = 17)	ATE PSG2 (n = 17)	ATE PSG3 (n = 17)	ATT PSG1 (n = 28)	ATT PSG2 (n = 28)	ATT PSG3 (n = 28)	Group diff PSG3 Mean Difference (95% CI)
**AHI**	13.6 (7.3)	2.4 (1.8)	1.1 (1.4)	14.4 (7.7)	3.3 (2.3)	0.8 (1.2)	0.3 (−0.5–1.1)
**OAHI**	12.3 (8.0)	1.2 (1.0)	0.6 (0.7)	12.6 (7.4)	1.3 (1.2)	0.5 (0.6)	0.1 (−0.3–0.5)
**ODI**	3.7 (4.0)	1.3 (1.1)	0.7 (0.5)	3.9 (3.9)	1.7 (1.7)	0.9 (1.1)	−0.2 (−0.8–0.4)
**RDI**	16.3 (7.2)	2.5 (2.0)	0.6 (0.7)	16.2 (7.8)	2.7 (2.3)	0.5 (0.6)	0.04 (−0.4–0.4)
**Mean SaO2 (%)**	96.5 (1.4)	97.0 (0.5)	96.7 (0.7)	96.7 (0.7)	97.1 (0.8)	96.6 (0.8)	0.06 (−0.4–0.5)
**Nadir O2 (%)**	87 (7.4)^a^	90.9 (3.5)^a^	91.1 (4.3)	86.4 (8.0)^a^	89.6 (5.0)	91.3 (4.3)	−0.1 (−2.8–2.6)

Data are mean (SD). Independent *t*-test was used. Group diff PSG3 = Difference between the groups in postoperative mean values at PSG3; ODI = oxygen desaturation index; RDI = respiratory disturbance index; SaO2 = oxygen saturation; ^a^ = missing data for less than 5 of the group participants.

The intention-to-treat using the LOCF resulted in a mean postoperative OAHI of 2.4 (SD 4.9) in the ATE group (n = 40), and 1.9 (SD 5.1) in the ATT group (n = 39). The mean difference between the groups postoperatively was −2.8 (CI -10.4–4.8). The simulative negative scenario for the ATT-group showed a mean difference of postoperative OAHI -0.2 (CI -2.5 – 2.0).

### 3.2 Secondary outcomes

The median OSA-18 decreased in the ATE group from 57 (IQR 47–79) to 27 (IQR 22–36) and in the ATT group from 67 (IQR 53–79) to 32 (IQR 25–44). No differences between the groups were seen for postoperative values of total OSA-18 score (p = 0.10), sleep disturbance (p = 0.18) or HRQoL (p = 0.17).

Five children operated with ATT were reoperated with ATE because of OSA relapse, one within six months of ATT (before PSG2), and the other four within 22 months. The sixth patient was reoperated because of recurrent tonsillitis after 36 months. No other child underwent any adenotonsillar surgery during the 5-year follow-up. The median age at first surgery was 34 months (IQR 30–37) for these six children, compared to 42 months (IQR 35–54) for the children who did not undergo a second surgery. Five of the reoperated children had a mean OAHI of 1 (SD 0.8) at five-year follow-up, and one did not attend the PSG3.

The dropout analysis revealed a significant difference in the distribution of surgical technique between children included and dropouts/excluded (Table 2); otherwise, the groups were similar in baseline characteristics.

## 4 Discussion

This study provides the long-term follow-up results of our previously published randomized controlled study that compared ATE with ATT in young children with moderate to severe OSA. The results indicate that ATT could be an effective treatment option for children with OSA. No group differences were found when assessing postoperative OAHI-values in either the per protocol (mean difference of 0.1, 95% CI -0.3 – 0.5) or sensitivity analyses, or quality of life with OSA-18 questionnaires. However, since the sample size was small, and there was a high dropout and exclusion rate of 43%, the results should be interpreted with caution.

The risk of OSA relapse after ATT has been reported in several studies with larger samples. For example, a prospective non-randomized two-year follow-up study by Vlastos and colleagues in 2008 reported that ATT with the cold steel scissor surgical technique (n = 243) resulted in reoperation among 3.5% of patients [22]. A review from 2015 presented a reoperation rate of second surgery between 0 and 11.9% [23]. In the Swedish National Patient Register, the reoperation rate after TT was 3.9%, 16.34 per 1000 person-years, within six years (median time 1.5 years) [8]. Of these, 82% were due to upper airway obstruction. The authors concluded that the risk for second surgery was higher among the youngest children undergoing TT (1–3 years). Further, that the follow-up time of six years is probably not long enough to cover the second peak of tonsil surgery (i.e., adolescence when many are operated on for recurrent tonsillitis) [24].

In the present study 6 of 39 (15%) children operated with ATT who were followed-up for 5 years were reoperated. Five of them within two years due to OSA relapse, and one within three years because of recurrent tonsillitis, giving a very high rate of 30.7 per 1000 person and year. A likely explanation could be the small sample size of young children with a known high risk of regrowth of the tonsil tissue. This was indicated by the low median age of 45 months in the ATT-group, and even lower median age of 34 months of the reoperated children. Further, since the children were followed up with PSG and the indication for revised surgery was set to an AHI of 5 or more, this may have resulted in a higher number of reoperations. In clinical practice, follow-ups and PSGs are rarely used, so there is a risk of missing the need for second surgery because of residual/recurrent OSA.

The strength of this study is the prospective randomized model, which minimizes the risks of selection bias and confounders. Furthermore, including young children between 2 and 6 years is rare. The fact that the children were evaluated with both PSG and OSA-18 at baseline, after one year and after five years is also a strength. Limitations of our study are the small groups and the dropout rate from PSG of 35%, which could be explained by the difficult procedure for a child and caregiver to undertake overnight in-laboratory PSG three times, especially if the child is considered recovered after surgery, from the caregivers’ aspect. Furthermore, this study may not be generalized to the overall population of children with OSA as it excluded patients with obesity and other comorbidities and risk factors for OSA. Another limitation is that we only studied one tonsillotomy technique (coblation), even though there are many other techniques (e.g., microdebrider, laser, cold steel, radiofrequency ablation) and, to diminish the risk of postoperative bleeding, we did not reduce tonsil mass deep into the fossae.

## 5 Conclusion

This long-term follow-up study of our previous RCT indicates that ATT could be effective in treating pediatric OSA after five years in young otherwise healthy children. The risk of relapse after ATT is notable, so information should be provided to caregivers, and follow-ups are necessary. Furthermore, the drop-out rate was high, and more extensive multi-center long-term studies are required.

## Authors' contributions

Dr Sjölander was involved in the data collection and analysis, interpreted the data, and wrote the manuscript.

Dr Borgström and Dr Nerfeldt were involved in the study design and the data collection and analysis and reviewed and revised the manuscript.

Prof Friberg conceptualized and designed the study, coordinated, and supervised data collection, reviewed and revised the manuscript.

All authors approved the final manuscript as submitted.

**Isabella Sjölander**: Investigation, Formal analysis, Writing- original draft preparation. **Anna Borgström**: Data curation, Investigation, Methodology, Writing – Reviewing and Editing. **Pia Nerfeldt**: Data curation, Investigation, Methodology, Writing – Reviewing and Editing. **Danielle Friberg**: Conceptualization, Methodology, Supervision, Project administration, Writing – Reviewing and Editing.

## Data sharing statement

This trial has been registered at www.clinicaltrials.gov (identifier NCT01676181). Deidentified individual participant data will not be made available.

## Funding/support

Uppsala County Council (ALF-project) and Acta-Otolaryngologica Foundation supported this research.

## Role of funder/sponsor

The funder/sponsor did not participate in the work.

## Conflicts of interest

The authors have no conflicts of interest that are relevant to this article.
